# The adult nasopharyngeal microbiome as a determinant of pneumococcal acquisition

**DOI:** 10.1186/2049-2618-2-44

**Published:** 2014-12-15

**Authors:** Amelieke JH Cremers, Aldert L Zomer, Jenna F Gritzfeld, Gerben Ferwerda, Sacha AFT van Hijum, Daniela M Ferreira, Joshua R Shak, Keith P Klugman, Jos Boekhorst, Harro M Timmerman, Marien I de Jonge, Stephen B Gordon, Peter WM Hermans

**Affiliations:** Laboratory of Pediatric Infectious Diseases, Department of Pediatrics, Radboudumc, 6500 HB Nijmegen, The Netherlands; Centre for Molecular and Biomolecular Informatics (CMBI) Bacterial Genomics, Radboudumc, 6500 HB Nijmegen, The Netherlands; Department of Clinical Sciences, Liverpool School of Tropical Medicine, L3 5QA Liverpool, UK; NIZO food research B.V., 6710 BA Ede, The Netherlands; Hubert Department of Global Health, Rollins School of Public Health, Emory University, Atlanta, GA 30322 USA; Crucell – Johnson & Johnson, Leiden, The Netherlands

**Keywords:** Experimental human model, Microbiome, Nasopharyngeal carriage, *Streptococcus pneumoniae*

## Abstract

**Background:**

Several cohort studies have indicated associations between *S. pneumoniae* and other microbes in the nasopharynx. To study causal relationships between the nasopharyngeal microbiome and pneumococcal carriage, we employed an experimental human pneumococcal carriage model. Healthy adult volunteers were assessed for pneumococcal carriage by culture of nasal wash samples (NWS). Those without natural pneumococcal carriage received an intranasal pneumococcal inoculation with serotype 6B or 23F. The composition of the nasopharyngeal microbiome was longitudinally studied by 16S rDNA pyrosequencing on NWS collected before and after challenge.

**Results:**

Among 40 selected volunteers, 10 were natural carriers and 30 were experimentally challenged. At baseline, five distinct nasopharyngeal microbiome profiles were identified. The phylogenetic distance between microbiomes of natural pneumococcal carriers was particularly large compared to non-carriers. A more diverse microbiome prior to inoculation was associated with the establishment of pneumococcal carriage. Perturbation of microbiome diversity upon pneumococcal challenge was strain specific. Shifts in microbiome profile occurred after pneumococcal exposure, and those volunteers who acquired carriage more often diverted from their original profile. *S. pneumoniae* was little prominent in the microbiome of pneumococcal carriers.

**Conclusion:**

Pneumococcal acquisition in healthy adults is more likely to occur in a diverse microbiome and appears to promote microbial heterogeneity.

**Electronic supplementary material:**

The online version of this article (doi:10.1186/2049-2618-2-44) contains supplementary material, which is available to authorized users.

## Background

*Streptococcus pneumoniae* (the pneumococcus) is one of the organisms that commensally reside in the human nasopharynx. Recently, an experimental human pneumococcal carriage model was established [1] which enables the controlled study of pneumococcal carriage episodes in the human nasopharyngeal niche. An episode of pneumococcal carriage is usually a beneficial immunogenic event [2], but it is also the potential initiation of invasive pneumococcal infections [3]. Pneumococcal carriage is most prevalent in children under 5, ranging from 10% up to 90% depending on specific age group and setting [4, 5], and is generally less prevalent in adults [6–12]. The incidence of invasive pneumococcal disease (IPD) is 10–20/100,000/year in developed countries [13–16] and is estimated to be even higher in developing countries [17], with those under 5 and over 50 years old most at risk. Annually, pneumonia causes an estimated 1.3 million childhood deaths worldwide [18]. The burden of IPD may be reduced by limiting the degree of pneumococcal carriage. Insight into the determinants of pneumococcal carriage will provide better understanding of how interference with this phenomenon could influence its prevalence. Known determinants of pneumococcal carriage are host traits related to exposure and immunity [4, 19, 20], antibiotic use, and pneumococcal vaccination [12]. An additional element that appears to be associated with pneumococcal carriage in children is the endogenous microbiota present in the nasopharyngeal cavity. For instance, it has been reported that in children, nasopharyngeal carriage of *S. pneumoniae* is inversely correlated with carriage of *S. aureus* [21–24]. Furthermore, investigation of the pediatric nasopharyngeal niche by a 16S rDNA sequencing approach has revealed associations between pneumococcal carriage and specific bacterial genera [25]. However, as these studies are cross-sectional, it is unknown how the endogenous nasopharyngeal microbiota modulates the establishment of pneumococcal carriage and what changes are brought about upon exposure to pneumococci. In this study, we compared the composition of the nasopharyngeal microbiome with and without naturally acquired pneumococcal carriage in healthy adults. In addition, a unique experimental human pneumococcal carriage model was employed to study whether specific compositions of the microbiota are associated with subsequent establishment of pneumococcal carriage and whether the adult nasopharyngeal microbiome is perturbed by exposure to *S. pneumoniae*.

## Results

### Selected volunteers and quality of samples

Among the 40 volunteers selected for microbiome analysis, 10 were natural carriers. The remaining 30 non-carriers were experimentally challenged. Pneumococcal carriage as detected by culture was established in 14 of the 26 selected volunteers who were inoculated with serotype 6B, and in 2 of the 4 challenged with serotype 23F. From these 40 volunteers, 117 samples were eligible for microbiome data analysis (Additional file 1: Table S1). The compositions of the individual 117 adult nasopharyngeal microbiomes are displayed in Additional file 2: Figure S1. The measured read count, richness, and diversity per sample were not influenced by the 16S rDNA concentration in the extracted DNA samples (Additional file 3: Figure S2).

### Distinct metagenomic profiles exist in the adult nasopharyngeal microbiome

**Figure 1:**
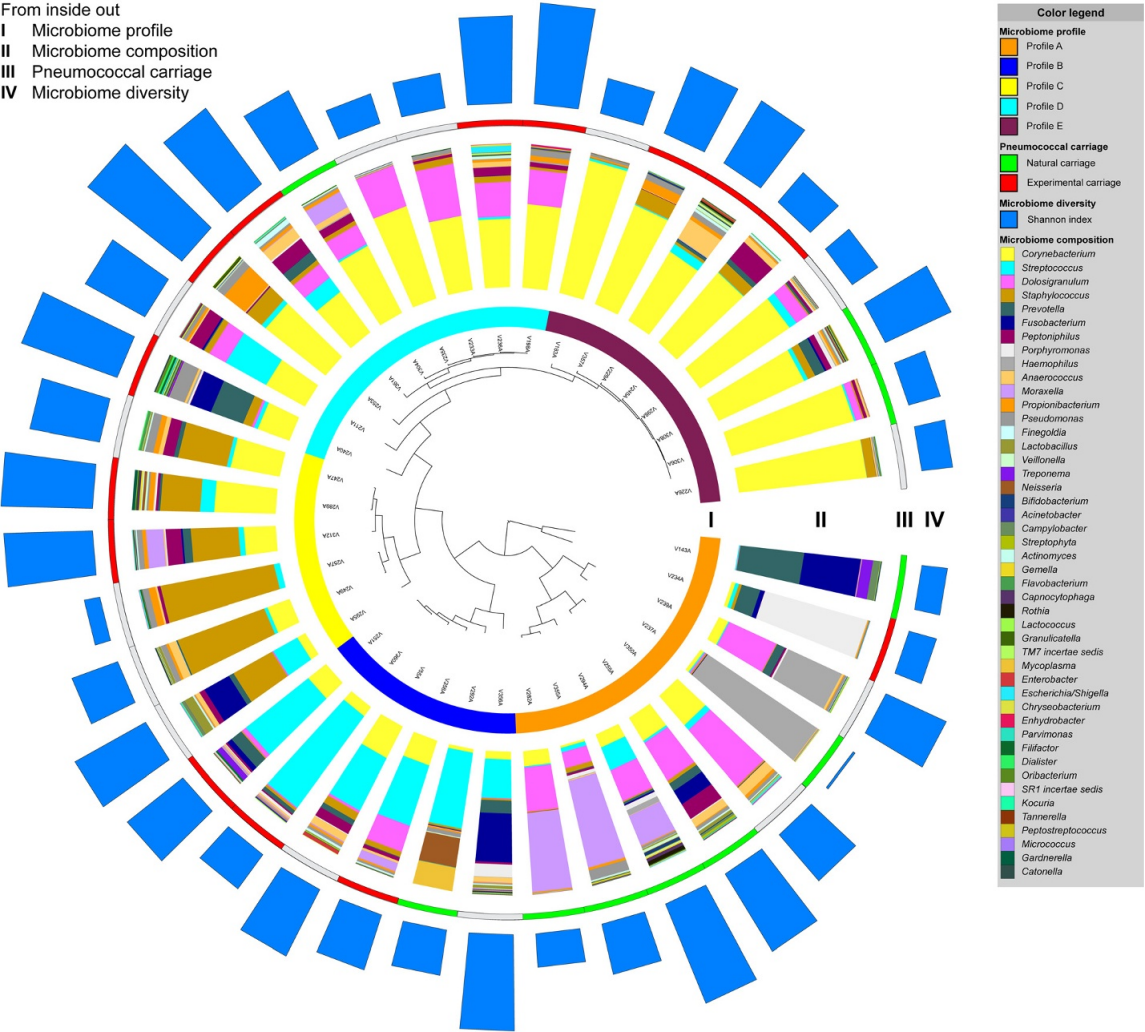
**Characteristics of the adult nasopharyngeal microbiome at baseline.** Each volunteer is represented by a branch of the circular hierarchical clustering tree. From inside out, at baseline, five distinct adult nasopharyngeal microbiome profiles were identified from the individual nasopharyngeal bacterial communities, appointed A to E (*I*). The individual microbiome compositions are proportionally displayed at genus level (*II*). Natural pneumococcal carriers are marked in *green*, experimentally colonized volunteers in *red*, and those without pneumococcal acquisition are depicted in *grey* (*III*). The length of the blue bars displays the diversity of the microbiome at baseline (*IV*). This figure was generated using iTOL [26].

**Figure 2:**
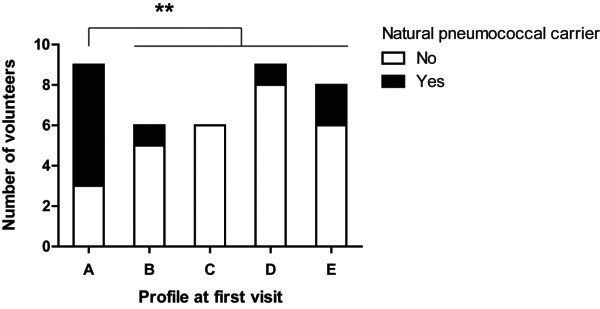
**Distribution of natural pneumococcal carriers over baseline microbiome profiles.** **: *p* <0.01.

The genera most frequently detected in the adult nasopharyngeal microbiome at baseline were *Corynebacterium*, *Dolosigranulum*, *Staphylococcus*, and *Streptococcus* (Additional file 4: Table S2). Starting from these most abundant genera, using hierarchical clustering and principal component analysis (PCA) on microbial densities of 155 operational taxonomic units (OTUs) present in the individual adult nasopharyngeal communities, five distinct microbiome profiles were identified and appointed as profile A to E (Figure 1). Corresponding PCA plots and principal coordinate analysis (PCoA) plots are displayed in Additional file 5: Figure S3. The random forest analysis showed a low (~15%) overall out of bag error between the class assignments of the samples, supporting the clustering analysis. In addition, the Pearson distances within profiles were lower compared with the distance between samples from different profiles (median (IQR): 0.25 (0.07–0.45) and 0.79 (0.51–0.95), respectively, *p* <0.0001). The most important taxa that allowed differentiation between the profiles were *Corynebacterium* spp., *Streptococcus* spp., *Staphylococcus* spp., *Dolosigranulum*, *Peptoniphilus*, and *Prevotella* as illustrated in Additional file 6: Figure S4. Among the five profiles, no differences in microbiome richness (number of different OTUs, *p* =0.92) or diversity (Shannon index, *p* =0.20; phylogenetic distance (PD) whole tree, *p* =0.53) were observed. Whereas the microbiomes of volunteers in profile B to E were all characterized by one or two specified OTUs, within profile A, the PDs between microbiomes were larger (unweighted UniFrac *p* =0.7, weighted UniFrac *p* <0.0001). Profile A was more frequently associated with natural pneumococcal carriage compared with the other profiles (*p* =0.0045) (Figure 2).

**Figure 3:**
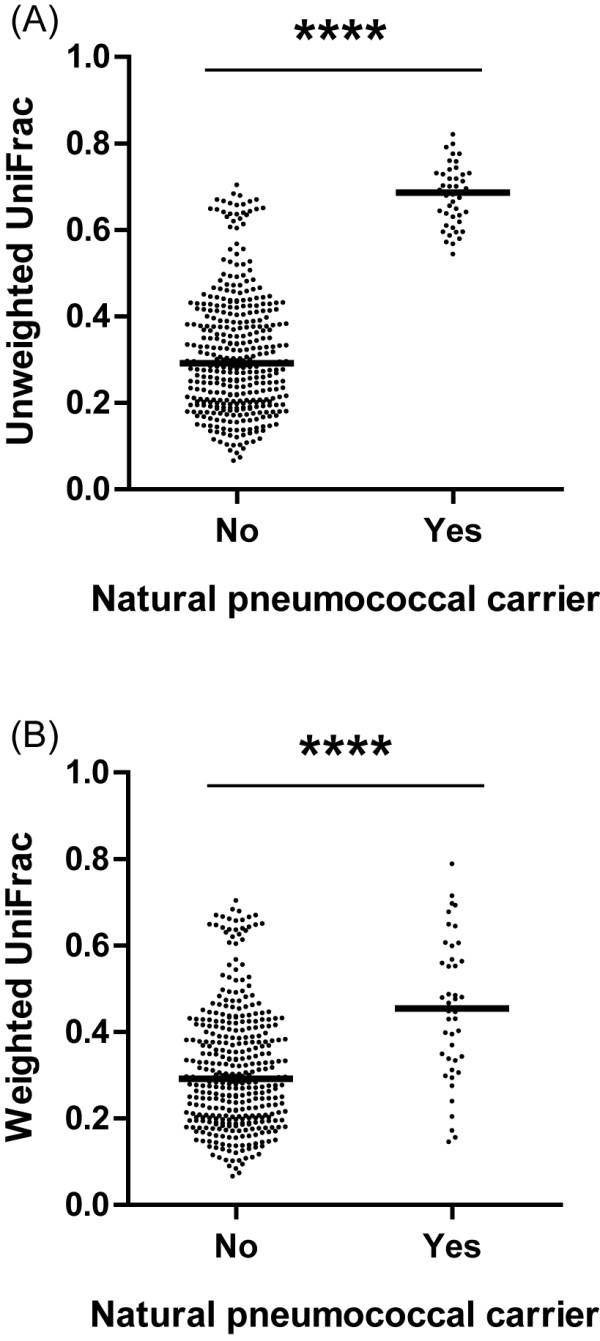
**Within-group phylogenetic distance of baseline microbiomes in volunteers with or without natural pneumococcal carriage.** Unweighted UniFrac **(A)** and weighted UniFrac **(B)**. ****: *p* <0.0001.

When comparing volunteers with and without natural pneumococcal carriage, no differences in microbiome richness (*p* =0.24) or diversity (Shannon index, *p* =0.36; PD whole tree, *p* =0.44) were observed. The PDs between the microbiomes of the natural pneumococcal carriers were larger compared with those in non-carriers (both unweighted and weighted UniFrac *p* <0.0001; Figure 3).

### The nasopharyngeal microbiome as a determinant of experimental pneumococcal carriage

**Figure 4:**
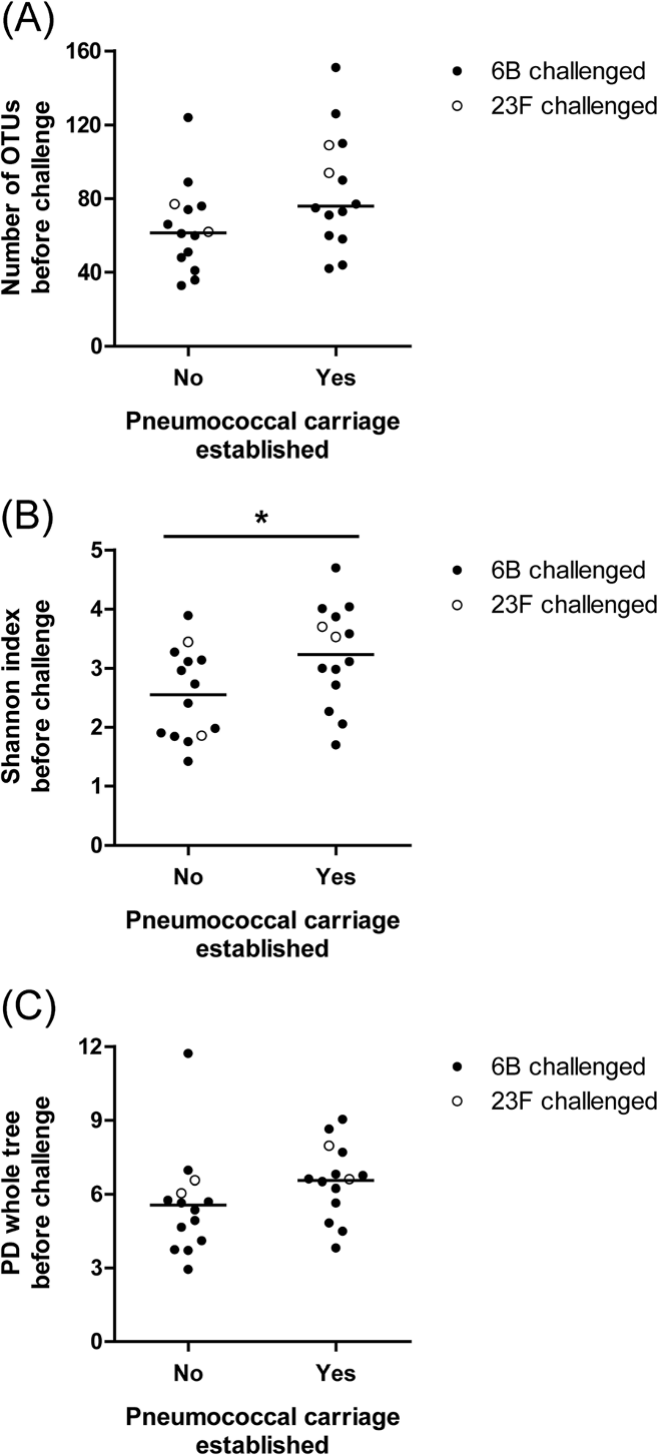
**Microbiome diversity prior to inoculation in volunteers with and without establishment of pneumococcal carriage.** Microbiome richness **(A)** diversity (Shannon index) **(B)** and PD whole tree, **(C)**. *: *p* <0.05.

The five microbiome profiles observed at baseline were not associated with successful pneumococcal carriage after inoculation with serotype 6B (Additional file 7: Figure S5), unlike the association observed for natural pneumococcal carriage. We did observe a positive association between a more diverse microbiome prior to challenge with serotype 6B or 23F (Shannon index 6B or 23F *p* =0.034; serotype 6B alone *p* =0.076) and successful experimental pneumococcal carriage (Figure 4). The relative abundance of specific OTUs as a determinant of pneumococcal carriage upon challenge with serotype 6B is displayed in Additional file 8: Figure S6. The *p* values of differences at OTU level were not significant after correction for multiple testing.

### Changes in microbiome profile are associated with successful pneumococcal carriage

Carriage of *S. pneumoniae* was not associated with a specific concurrent microbiome profile in the first 2 weeks after challenge (Additional file 9: Figure S7). The fraction of volunteers shifting to a different microbiome profile was stable over time (44%, 46%, and 48%, respectively, *p* =1), so the time from challenge did not affect the number of shifts. Volunteers who acquired pneumococcal carriage less often returned to their original microbiome profile at 14 days post challenge (5/13 versus 12/14, *p* =0.018). None of the pneumococcal carriers had turned to the *Staphylococcus*-dominant microbiome profile C, despite the fact that microbiome profile C was involved in profile shifts, as divergence from and convergence to profile C did not differ from rates in other profiles (44% versus 45%, *p* =1, and 38% versus 47%, *p* =0.6, respectively).

### Perturbations in microbiome diversity appear to be strain dependent

**Figure 5:**
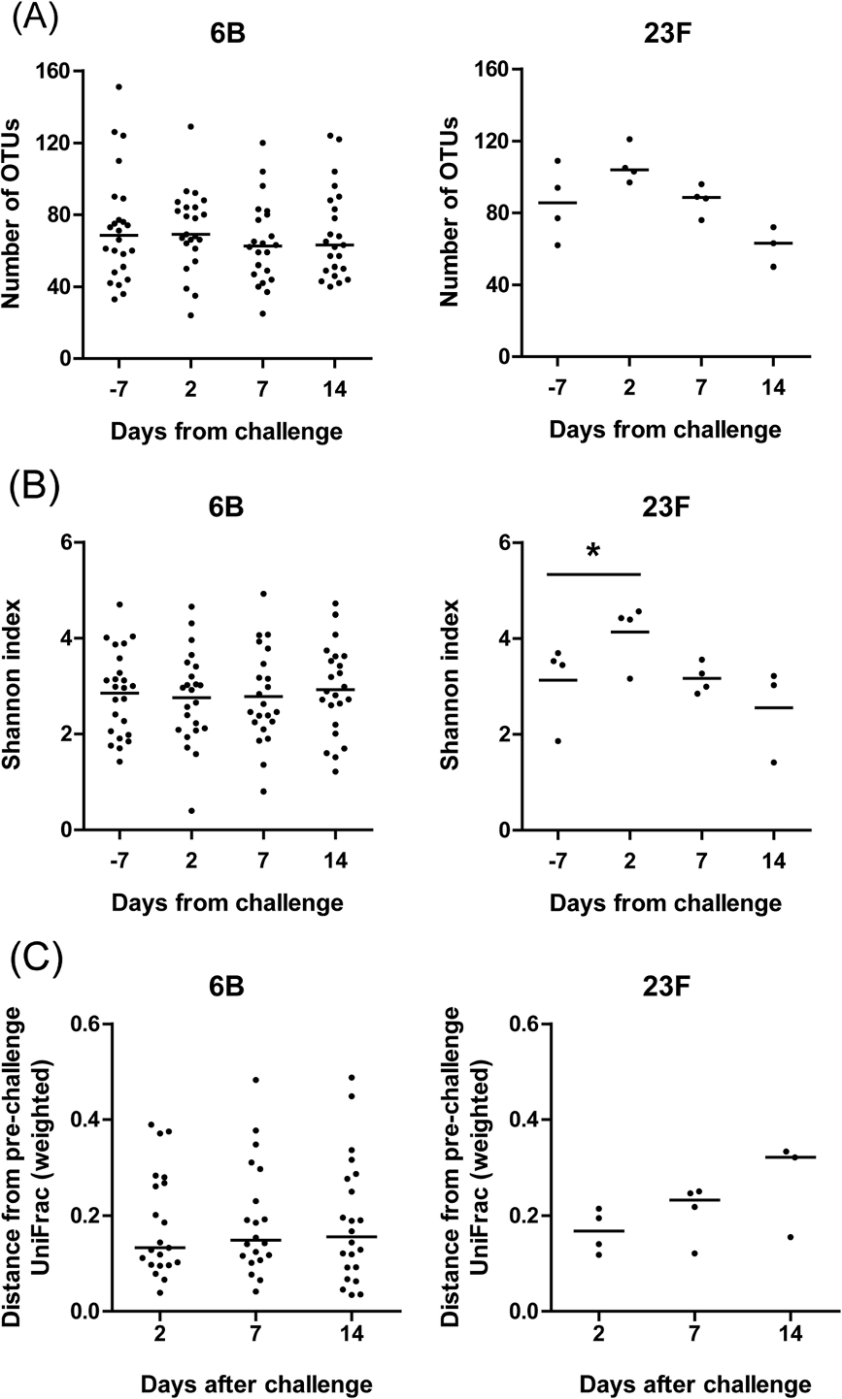
**Perturbations in the nasopharyngeal microbiome after pneumococcal challenge.** Microbiome richness **(A)** and diversity **(B)** and its phylogenetic divergence from the microbiome prior to challenge **(C)**. *6B* inoculated with serotype 6B, *23F* inoculated with serotype 23F; *: *p* <0.05.

Intranasal inoculation with a serotype 6B strain was not associated with consistent perturbations in the nasopharyngeal microbiome with respect to richness, diversity, and PD from the composition prior to challenge. In contrast, inoculation with a serotype 23F strain was associated with a transient increase in diversity in all volunteers (Shannon index: increase from day -7 to 2, *p* =0.004; decrease from day 2 to 7, *p* =0.083; Figure 5). The observed transient increase in richness was not significant (increase from day -7 to 2, *p* =0.25; decrease from day 2 to 7, *p* =0.13), indicating that not only an increase in the number of different OTUs occurred after challenge but also equalization of the relative abundance of different OTUs. The PD to the microbiome prior to inoculation with serotype 23F increased from day 2 to day 7 in all volunteers but was not statistically significant (weighted UniFrac *p* =0.13). Differences in relative abundance of specific OTUs after pneumococcal challenge with serotype 6B are displayed in Additional file 10: Figure S8. The *p* values of differences at OTU level were not significant after correction for multiple testing.

## Discussion

We longitudinally studied the composition of the nasopharyngeal microbiome in healthy adults before and after experimental pneumococcal challenge. At baseline, five nasopharyngeal microbiome profiles were identified with different natural pneumococcal carriage rates. The PD between microbiomes of natural carriers was particularly large compared with that of non-carriers. A more diverse microbiome prior to challenge was associated with subsequent establishment of pneumococcal carriage. Those volunteers who acquired carriage more often diverted from their original microbiome profile than non-carriers. Furthermore, perturbation of microbiome diversity upon exposure to pneumococci appeared to be strain dependent.

The unique experimental human pneumococcal carriage model mimics natural colonization and allows discrimination between microbial determinants and consequences of pneumococcal carriage. Rapid processing of samples ensured a high DNA yield for accurate microbiome data. Pneumococcal carriage was assessed by culture—the gold standard—which has been demonstrated to be a robust method in nasal wash samples [27]. Based on 97% read identity after pyrosequencing of the V3-V6 region of the 16S rDNA gene, it was not possible to differentiate between *S. pneumoniae* and other streptococci relevant to the nasopharyngeal niche (i.e., *S. mitis*, *S. mutans*, and *S. pseudopneumoniae*). Although it may be possible to confirm pneumococcal carriage by sequencing the V1-3 region [28], V3-V6 was selected as it ensures high coverage and high phylogenetic resolution for a universal approach in a niche of interest.

The relatively small number of volunteers impeded studying the involvement of specific OTUs in pneumococcal carriage. Volunteers inoculated with different doses could be grouped, because the applied doses conferred similar rates and densities of pneumococcal carriage [2]. The unequal group sizes per inoculation strain were due to a difference in colonization efficiency between the two strains applied (Gritzfeld and coworkers, submitted for publication). Another limitation was the inability to control other environmental influences like natural exposure and viral infection. Inherent to the model, those volunteers most at risk for acquiring pneumococcal carriage may actually have been the natural carriers who were not inoculated.

In this study, clustering of individual microbial communities into microbiome profiles was performed without weighting of phylogenetical distances between OTUs, because phylogenetically closely related OTUs may still show distinct patterns in the human nasopharyngeal niche. Compared with four highly robust microbiome profiles, profile A contained more heterogeneous microbiomes and may therefore hold subprofiles not identified by this study. Although a similar grouping of bacteria was observed in sampling from the anterior nares in the Human Microbiome Project [29], already within the nasal cavity, three distinct epithelium type-specific microbiota have been identified [30]. Therefore, it seems inappropriate to directly compare the identified microbiome profiles in the nasopharynx—habitat to the pneumococcus—to those in other nasal sites. Consistent with a study that specifically investigated the nasopharyngeal niche in healthy Chinese undergraduates [31], the top four most abundant bacterial phyla present in the adult nasopharyngeal microbiome at baseline were Actinobacteria, Fimicutes, Proteobacteria, and Bacteroidetes. The same holds true for the top three most abundant genera observed: *Corynebacterium*, *Dolosigranulum*, and *Staphylococcus. Streptococcus* ranked fourth in our study, but it was not mentioned in the top nine reported by the Chinese study. This may be explained by differences in our study population (i.e., more volunteers, slight overrepresentation of natural carriers, and distinct geographical region) or the use of different nasopharyngeal sampling methods.

The adult nasopharyngeal microbiome differed from that in children. At phylum level, we observed an increased abundance of Actinobacteria (3% in children, >30% in adults) and a decreased abundance of Proteobacteria (64% in children, <15% in adults) [31, 32]. Whereas pneumococcal carriage in children has previously been associated with a high level of *Streptococci* in the nasopharynx and a low diverse concurrent microbiome [25, 33], in our adult volunteers, natural pneumococcal carriage was associated with low proportions of streptococcal reads next to normally diverse and phylogenetically heterogeneous microbiome compositions. This may indicate that, in contrast to children, in adults, the pneumococcus generally does not predominate in the nasopharyngeal microbial community upon acquisition.

We observed that a more diverse microbiome prior to challenge was associated with the establishment of pneumococcal carriage when combining the data from both 23F- and 6B-inoculated volunteers. We have noted that there may be strain-specific effects on the microbiome composition following successful colonization, and therefore, we cannot exclude strain-specific microbiome diversity requirements for successful colonization. However, the decrease in *p* value while combining the 23F and 6B data suggests that this is not the case, making pooling a valid approach. Although in other host-associated microbial communities a more diverse microbiota was found to be associated with resistance to pathogens, we did not observe this phenomenon in our study. Here, a more diverse nasopharyngeal microbiome appears to facilitate pneumococcal carriage. As pneumococcal carriage is proposed to be an immunizing event [2], we speculate that short-term low-level pneumococcal carriage actually promotes health as this will boost protective immunity against the pneumococcus.

None of the volunteers with the *Staphylococcus*-dominated microbiome profile C acquired pneumococcal carriage, and none of the pneumococcal carriers turned to profile C. This observation is in line with the frequently described inverse correlation between carriage of *S. pneumoniae* and *S. aureus* in both children and adults [11, 21–24], although we cannot exclude that a different *Staphylococcus* species is responsible for the observed phenomenon in our case. After pneumococcal challenge, we observed continuous shifts in microbiome profiles, although volunteers without pneumococcal acquisition more often returned to their original profile. Stability in the upper respiratory tract microbiome has been noted before by Charlson et al., where the nasopharyngeal community composition was less robust over time compared to the microbiome in the oropharynx, but remained relatively stable [34]. Furthermore, whereas challenge with serotype 6B was not associated with specific changes in microbiome diversity, challenge with serotype 23F was. As the 23F-inoculated volunteers were primarily staphylococcal carriers, this may have influenced the changes observed. However, the increased variability after challenge with 23F was not observed in other volunteers with *Staphylococcus*-dominated profiles who were challenged with 6B. Therefore, we suggest that perturbations in microbiome diversity after pneumococcal exposure may be strain dependent.

## Conclusions

In healthy adults, the nasopharyngeal microbiome can be subdivided in at least five bacterial community classes having at least four distinct profiles, with profiles B–E dominated by *Streptococcus* spp., *Staphylococcus* spp., *Corynebacterium* spp., or a combination of *Corynebacterium* spp. and *Dolosigranulum*, whereas profile A is more diverse. There is evidence of an inverse correlation between carriage of *S. pneumoniae* and *S. aureus*. In our study, a more diverse microbiome was associated with the establishment of pneumococcal carriage. Furthermore, *S. pneumoniae* was generally little prominent in carriers and its acquisition appears to promote microbial heterogeneity. Whether pneumococcal carriage in healthy adults is a prosperous event besides one related to disease remains to be elucidated.

## Methods

### Experimental study model

We performed the experimental human pneumococcal carriage model as previously described [1]. In short, healthy adult volunteers were recruited in the United Kingdom according to inclusion and exclusion criteria described [1]. At baseline, we collected a nasal wash sample (NWS) and assessed pneumococcal carriage by culture of the NWS specimen. Of the 40 volunteers included for microbiome analysis, 10 were natural carriers. Those volunteers who were not natural pneumococcal carriers were inoculated with either a serotype 6B strain (BHN418 [35]) or 23F strain (P833 [36]) at 60,000- to 320,000-colony-forming-unit (CFU) *S. pneumoniae* as these generally have a low invasiveness rate and have been successfully used in previous carriage studies [1, 35]. Follow-up NWS were collected at 2, 7, and 14 days after challenge. Acquisition of pneumococcal carriage with the inoculated strain was determined by culture and confirmed by latex agglutination serotyping of recovered strain. This study has been conducted according to the Declaration of Helsinki principles, and ethical approval was obtained from the National Health Service Research Ethics Committee, Sefton, Liverpool (11/NW/0592). Written informed consent was received from all volunteers prior to inclusion in the study.

### Bacterial DNA extraction

Directly after NWS collection, we mixed 2 ml of the sample with 4 ml RNAprotect Bacteria Reagent (cat. no. 76506, Qiagen, Venlo, The Netherlands) to precipitate and protect nucleic acids in the sample. After 5 min of incubation at room temperature (RT), the sample was stored at -80°C until further use. The thawed 6-ml suspension was pelleted in a 2-ml tube by three centrifugation steps, each for 20 min at 13,200 rpm at 4°C in a microcentrifuge (cat. no. 5415R, Eppendorf, Hamburg, Germany). To the pellet, we added 0.3 ml lysis buffer with protease (Agowa Mag mini DNA extraction kit, cat. no. NAP40401, LGC Genomics, Berlin, Germany), 50 mg sterilized zirconia/silica beads (diameter 0.1 mm, cat. no. 11079101z, BioSpec Products, Bartlesville, OK, USA), and 0.3 ml phenol (Phenol BioUltra, cat. no. 77607, Sigma-Aldrich, St. Louis, MO, USA). The sample was mechanically disrupted by bead beating in a TissueLyser LT (cat. no. 85600, Qiagen, Venlo, The Netherlands) for two times 2 min at 50 Hz, cooling the sample on ice after each step. We centrifuged the homogenate for 10 min at 10,000 rpm at RT and transferred the aqueous phase to a 1.5-ml tube. After addition of a binding buffer (twice the aqueous phase volume) and 10 μl of magnetic beads, the sample was incubated for 30 min at RT in a mixing machine. We washed the magnetic beads with 200 μl of each wash buffer 1 and 2 and eluted the DNA with 63 μl elution buffer according to the manufacturer’s instructions.

### Bacterial DNA quantification and sample selection

We determined the bacterial DNA concentration in each eluate by qPCR on the 16S rDNA gene. The primer and probe sequences were as follows: forward primer 5′-CGA AAG CGT GGG GAG CAA A-3′; reverse primer 5′-GTT CGT ACT CCC CAG GCG G-3′; probe 5′-(FAM)-ATT AGA TAC CCT GGT AGT CCA-(MGB)-3′ as previously published by Bogaert et al. [32]. The 25-μl PCR mix was 1× TaqMan Universal PCR Master Mix, 10 μM of each primer (1 μl), 5 μM probe (1 μl), 6.5 μl DNA-free water, and 3 μl template DNA. Thermal cycling was performed in a ABI 7500 Fast Real-Time PCR System (cat. no. 4351107, Life Technologies, Carlsbad, CA, USA), with the following cycling conditions: 2 min 50°C, 10 min 95°C, and 50 cycles of 15 s at 95°C and 1 min at 65°C. The 16S rDNA standard curve consisted of a 10-fold dilution series of a mix of genomic DNA extracted from three bacteria common to the respiratory tract: *Streptococcus pneumoniae* (TIGR4), *Moraxella catarrhalis* (RH4), and *Haemophilus influenzae* (1521062). We extracted genomic DNA with the Qiagen Genomic-tip 20/G Kit (cat. no. 10223, Qiagen, Venlo, The Netherlands) and quantified it by a spectrophotometer (NanoDrop ND-1000, Thermo Fisher Scientific, Wilmington, DE, USA). Those volunteers whose extracted DNA samples all contained at least 1 pg bacterial DNA/μl [37] were considered eligible for microbiome analysis. To avoid false-positive results, both DNA extraction and amplification procedures were accompanied by negative controls.

### 16S rDNA pyrosequencing and handling of DNA sequences

We amplified the V3-V6 region of the 16S rDNA gene with forward primer 5′*-CCA TCT CAT CCC TGC GTG TCT CCG ACT CAG NNNNNN***ACT CCT ACG GGA GGC AGC AG**-3′ (italicized sequence: 454 Life Sciences primer A; bold sequence: broadly conserved bacterial primer 338 F; *NNNNNN:* the sample-specific six-base barcode used to tag each PCR product) and reverse primer 5′-*CCT ATC CCC TGT GTG CCT TGG CAG TCT CAG***CRR CAC GAG CTG ACG AC**-3′ (italicized sequence: 454 Life Sciences primer B; bold sequence: broadly conserved bacterial primer 1061R). We purified the amplicons from the PCR product using two kits consecutively according to their manufacturer’s instructions: the MSB Spin PCRapace Kit (cat. no. 1020220400, Isogen, De Meern, The Netherlands) and the PureLink Quick PCR Purification Kit (cat. no. K310002, Life Technologies, Bleiswijk, The Netherlands) using binding buffer 3B and an elution volume of 40 μl. A composite sample for pyrosequencing was prepared by pooling 100 ng purified PCR product from each sample. Fifty microliters of the amplicon library (concentration 14.5 ng/μl) was submitted for pyrosequencing on the 454 Life Sciences GS-FLX + platform using Titanium sequencing chemistry (both Roche, Germany) at GATC Biotech, Konstanz, Germany. One hundred seventeen samples were analyzed (Additional file 1: Table S1).

We analyzed the pyrosequencing data with a workflow based on QIIME v1.2 [38], using settings as recommended in the QIIME 1.2 tutorial, with the following exceptions: reads were filtered for chimeric sequences using ChimeraSlayer [39], and OTU clustering was performed with settings as recommended in the QIIME newsletter 2010 [40]. The RDP classifier version 2.2 was performed for taxonomic classification [41]. Samples with read counts below 500 were excluded from further analysis. Sequence data and subject characteristics are available at http://www.cmbi.ru.nl/bamics/supplementary/cremers_2014_ehpc/index.htm.

### Diversity estimates, correlation, and clustering analysis

To correct for differences in read count while calculating diversity estimates, individual sample data were down-sampled to the lowest read count included in the study. We measured alpha diversity within samples by richness (number of OTUs) and two diversity estimates: the Shannon index that increases with OTU number and with equality of OTU abundances, and the PD whole tree which accentuates phylogenetically distant OTUs. We measured beta diversity between samples by the UniFrac distance that estimates the fraction of a sample’s phylogenetic tree that differs from another sample, with (weighted) or without (unweighted) emphasis on the most abundant OTUs. Starting from the most abundant genera present in the nasopharynx at baseline, we studied the presence of microbiome profiles using hierarchical clustering by Pearson correlations and PCA on microbial density expressed as the percentage of reads in a sample that is assigned to a specific OTU, where an OTU should account for at least 1% of the reads in one sample. PCA on OTU abundance data and PCoA on weighted and unweighted UniFrac distances were performed in R using prcomp and labDSV [42], respectively, with default settings. Random forest analysis with microbiome profiles as classes and the microbial density data as classifiers was performed using the randomForest package in Bioconductor [43]. In addition, we compared Pearson distances within profiles to those between profiles. The microbial communities observed in samples collected after inoculation were allocated to a microbiome profile using random forest analysis handling microbial density data as described above. Correlation analysis between OTUs was performed by pairwise Spearman’s correlations.

### Statistical analyses

Differences in dichotomous variables (pneumococcal carriage) were statistically tested by a Fisher’s exact test. Normality of the distribution of continuous variables was tested by Shapiro-Wilk test. Differences in microbiome characteristics by normally distributed variables (Shannon index, PD whole tree) were statistically tested by an unpaired *t*-test for two independent groups or a one-way analysis of variance (ANOVA) for multiple independent groups and by a paired *t*-test or repeated-measures one-way ANOVA for perturbations in the microbiome over time. Differences in not normally distributed variables (number of OTUs, Pearson distance, UniFrac) were statistically tested by a Mann-Whitney *U* test for two independent groups or a Kruskal-Wallis test for multiple independent groups. To test perturbations in the microbiome over time, a Wilcoxon matched-pairs signed rank test or Friedman test was applied. For all analyses, the significance level was set at 0.05. Multiple testing correction was performed by the Benjamini-Hochberg false discovery rate procedure [44].

## Availability of supporting data

Sequence data and subject characteristics are available at http://www.cmbi.ru.nl/bamics/supplementary/cremers_2014_ehpc/index.htm.

## Electronic supplementary material

Additional file 1: Table S1: Samples selected for microbiome analysis and their bacterial DNA concentrations by 16S rDNA qPCR (pg/μl). (DOCX 21 KB)

Additional file 2: Figure S1: Microbiome composition of the 117 individual nasopharyngeal samples represented at genus level. Positive pneumococcal culture results are displayed on the left (green: natural carrier, red: carriage of inoculation strain) and their individual diversity metrics on the right. (TIFF 16 MB)

Additional file 3: Figure S2: Correlation between 16S rDNA quantity and read count, richness, and diversity. The 16S rDNA concentration in the extracted DNA samples has not influenced the sequencing yield in terms of the number of reads (*p* =0.074) (panel A), the number of OTUs (*p* =0.64) (panel B), or the Shannon index (*p* =0.14) (panel C) per sample. (TIFF 9 MB)

Additional file 4: Table S2: Most prevalent bacterial genera present in the adult nasopharynx at baseline by natural carriage status. (DOCX 16 KB)

Additional file 5: Figure S3: PCA and PCoA plots of the individual nasopharyngeal microbial communities at baseline. *PCA* principal component analysis, *PCoA* principal coordinate analysis. Individual volunteers are displayed as dots colored according to microbiome profile, in two-dimensional graphs with combinations of the three PCA components on the axes (panel A). PCoA was performed on unweighted and weighted UniFrac distances (panel B). (TIFF 14 MB)

Additional file 6: Figure S4: The percentage of reads from each OTU that differentiates between the microbiome profiles per NWS. *NWS* nasal wash sample. (TIFF 8 MB)

Additional file 7: Figure S5: Distribution of experimental pneumococcal carriers over the five nasopharyngeal microbiome profiles at baseline. Volunteers who received a pneumococcal challenge with serotype 6B are displayed. (TIFF 7 MB)

Additional file 8: Figure S6: OTU abundances at baseline in volunteers with or without acquisition of experimental pneumococcal carriage. Volunteers who received a pneumococcal challenge with serotype 6B are displayed. Nodes represent taxa, and edges link the different taxonomic levels. The fold increase is calculated as the log_2_ of the ratio of the relative abundance in pre-existing microbiome compositions of volunteers without and with establishment of pneumococcal carriage after challenge (0 = no difference between those who did and did not establish carriage, 1 = twice as abundant in those who established carriage, and so on). The significance is expressed as the *p* value of a Mann-Whitney *U* test of the baseline samples from all challenged volunteers. Note that the relation between node size and total abundance is non-linear. (TIFF 2 MB)

Additional file 9: Figure S7: Distribution of pneumococcal culture-positive samples after challenge with serotype 6B over the microbiome profiles. (TIFF 7 MB)

Additional file 10: Figure S8: Changes from baseline OTU abundances 2 days after pneumococcal challenge among serotype 6B-challenged volunteers. Nodes represent taxa, and edges link the different taxonomic levels. The fold increase is calculated as the log_2_ of the ratio of the relative abundance in samples before and 2 days after pneumococcal challenge (0 = no difference between before and after challenge, 1 = twice as abundant after challenge, and so on). The significance is expressed as the *p* value of a Mann-Whitney *U* test of the samples before and 2 days after challenge. Note that the relation between node size and total abundance is non-linear. (TIFF 309 KB)
