# Supplementary material for: The adult nasopharyngeal microbiome as a determinant of pneumococcal acquisition
Source: Microbiome. 2014 Dec 15;2:44. doi: 10.1186/2049-2618-2-44 (PMC4323220; doi:10.1186/2049-2618-2-44)
Supplement: Supplementary file 1 — Additional file 1: Table S1: Samples selected for microbiome analysis and their bacterial DNA concentrations by 16S rDNA qPCR (pg/μl). (DOCX 21 KB) [file 40168_2014_72_MOESM1_ESM.docx]

**Table S1. Samples selected for microbiome analysis and their bacterial DNA concentrations by 16S rDNA qPCR (pg/µl).**

|  | **Time point (days from challenge)** | | | |  |
| --- | --- | --- | --- | --- | --- |
| **Volunteer** | **-7 days** | **2 days** | **7 days** | **14 days** |  |
| **6B inoculated (n=26)** | | | | |  |
| 198/13 | 2.0 | 4.5 | 4.1 | 10.3 | **14 carriers** |
| 236/52 | 6.4 | 6.7 | 3.9 | 10.1 |  |
| 240/56 | 1.3 | 24.1 | 13.1 | 7.8 |  |
| 245/62 | 13.3 |  | 12.3 | 11.8 |  |
| 251/68 | 12.3 |  | 1.3 | 25.6 |  |
| 360/170 | 1.1 | 6.0 | 2.8 | 4.5 |  |
| 361/171 | 6.0 | 12.1 | 2.7 | 9.6 |  |
| 234/50 | 1.9 | 2.9 | 4.1 | 2.2^A^ |  |
| 307/119 | 1.5^B^ | 3.8 | 14.2 |  |  |
| 354/161 |  | 4.9 | 2.8 | 5.6 |  |
| 229/45 | 1.2 | 2.9 | 7.7 | 1.5 |  |
| 253/69 | 2.9 | 8.8 | 2.5 | 1.2 |  |
| 358/166 | 3.6 | 6.4 | 3.7 | 3.4 |  |
| 357/165 | 1.5 | 2.0 |  | 1.2 |  |
| 235/51 | 1.3 | 1.8 | 1.6 | 1.7 | **12 non carriers** |
| 183/164 | 2.0 | 1.5 | 1.2 | 1.9 |  |
| 211/26 | 2.3 | 8.8 | 3.6 | 4.9 |  |
| 233/49 | 1.3 | 5.5 | 7.1^A^ | 19.9 |  |
| 350/160 | 4.3 | 2.7 | 3.8 | 1.8 |  |
| 55/168 | 6.0 | 6.5 | 14.3 | 12.1 |  |
| 226/42 | 2.8 |  |  | 3.7 |  |
| 239/55 | 13.8 | 10.2 | 60.8 | 15.6 |  |
| 247/64 | 1.3 | 6.1 |  | 2.3 |  |
| 249/66 | 1.6 | 1.3 | 1.5 | 1.1 |  |
| 257/72 | 2.3 | 1.7 | 1.9 | 3.4^A^ |  |
| 356/163 | 18.9 | 51.5 | 5.6 | 87.1 |  |
|  |  |  |  |  |  |
|  | | | | |  |
| **23F inoculated (n=4)** | | | | |  |
| 289/101 | 8.2 | 11.6 | 13.0 | 31.4 | **2 carriers** |
| 312/123 | 4.8 | 3.6 | 10.9 | 12.8 |  |
| 295/108 | 28.8 | 15.9 | 10.0 | 26.9 | **2 non carriers** |
| 298/111 | 10.4 | 20.2 | 2.6 | 20.0^A^ |  |
|  |  |  |  |  |  |
|  | | | | |  |
| **Natural carriers, not inoculated (n=10)** | | | | | **Serotypes detected** |
| 255/167 | 3.0 | 2.2 | 2.2 | 1.8 | 33 |
| 355/162 | 2.5 | 1.5 | 3.3 | 9.1 | 19 |
| 143/59 | 68.3 |  |  |  | 11 |
| 237/53 | 30.5 |  |  |  | 15 |
| 282/95 | 53.2 |  |  |  | 9 |
| 292/105 | 15.0 |  |  |  | 3 |
| 294/107 | 7.0 |  |  |  | 3 |
| 304/116 | 7.2 |  |  |  | 15 |
| 306/118 | 10.9 |  |  |  | NVT |
| 308/120 | 7.1 |  |  |  | 3 |
|  |  |  |  |  |  |
|  |  | | | | |
|  | Culture of nasal wash positive for *S. pneumoniae* | | | | |
| NVT: non vaccine type | | | | | |
| ^A^Sample yielded too little 16S rDNA amplicon for sequencing | | | | | |
| ^B^Read count below 500 after sequencing | | | | | |
