# Supplementary figures and images for: The adult nasopharyngeal microbiome as a determinant of pneumococcal acquisition
Source: Microbiome. 2014 Dec 15;2:44. doi: 10.1186/2049-2618-2-44 (PMC4323220; doi:10.1186/2049-2618-2-44)

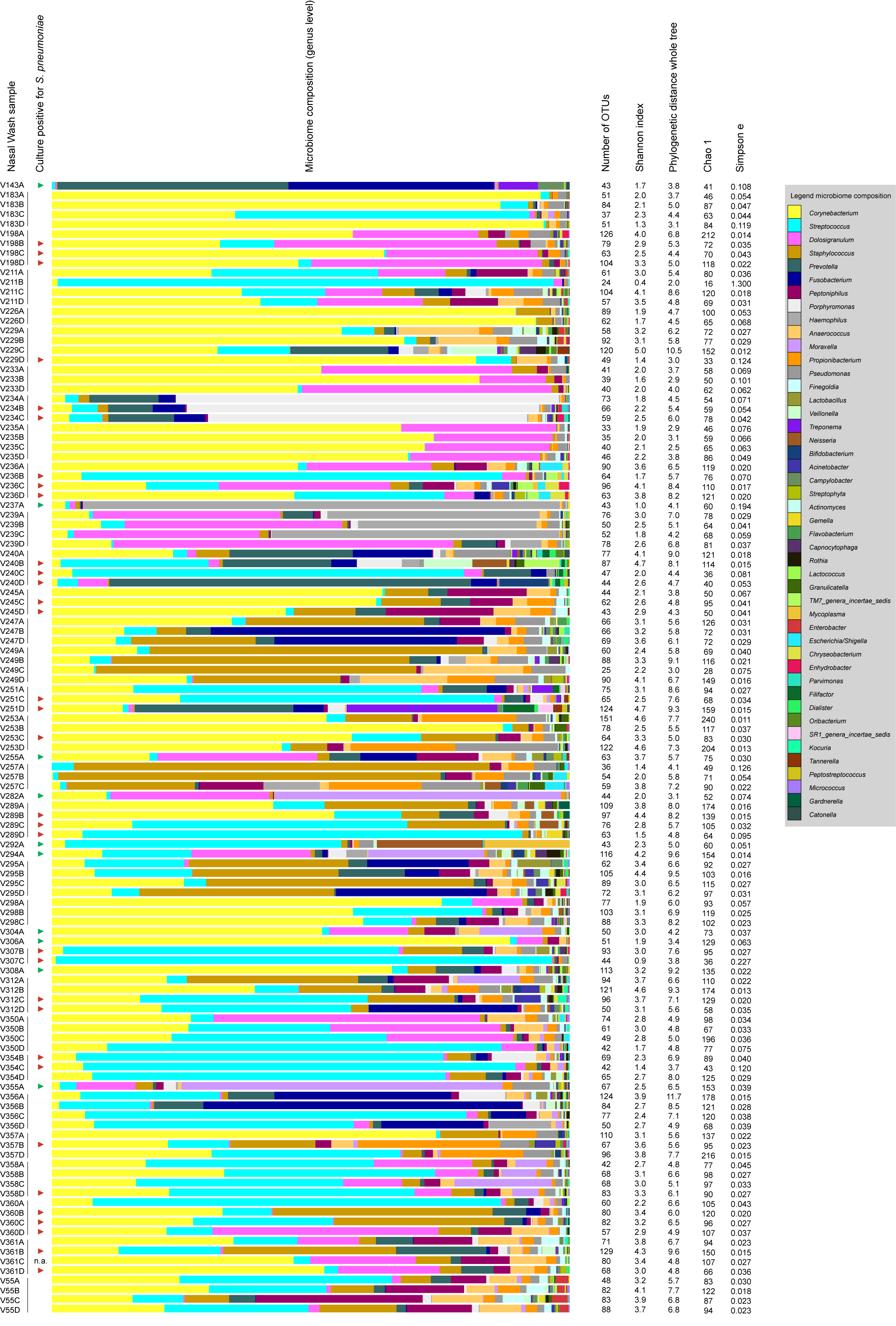

Supplement: Supplementary file 2 — Additional file 2: Figure S1: Microbiome composition of the 117 individual nasopharyngeal samples represented at genus level. Positive pneumococcal culture results are displayed on the left (green: natural carrier, red: carriage of inoculation strain) and their individual diversity metrics on the right. (TIFF 16 MB) [file 40168_2014_72_MOESM2_ESM.tiff]

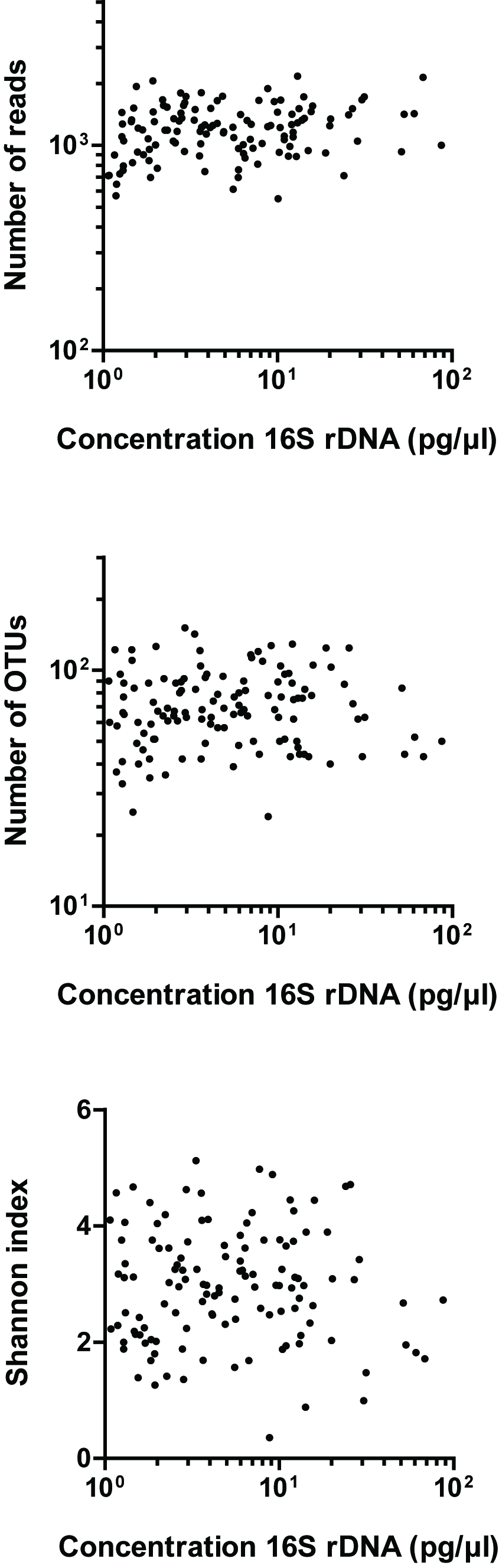

Supplement: Supplementary file 3 — Additional file 3: Figure S2: Correlation between 16S rDNA quantity and read count, richness, and diversity. The 16S rDNA concentration in the extracted DNA samples has not influenced the sequencing yield in terms of the number of reads (p =0.074) (panel A), the number of OTUs (p =0.64) (panel B), or the Shannon index (p =0.14) (panel C) per sample. (TIFF 9 MB) [file 40168_2014_72_MOESM3_ESM.tiff]

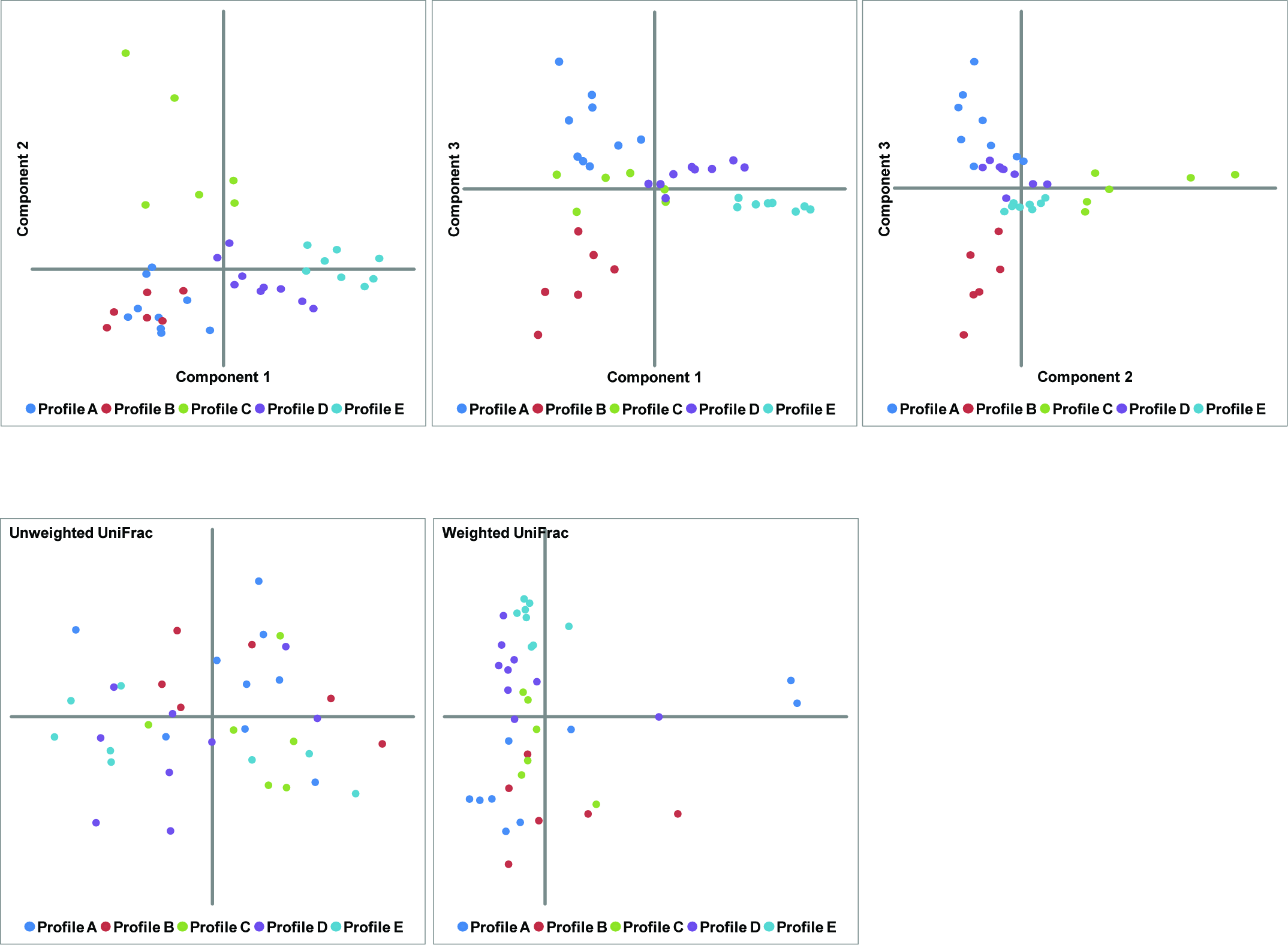

Supplement: Supplementary file 5 — Additional file 5: Figure S3: PCA and PCoA plots of the individual nasopharyngeal microbial communities at baseline. PCA principal component analysis, PCoA principal coordinate analysis. Individual volunteers are displayed as dots colored according to microbiome profile, in two-dimensional graphs with combinations of the three PCA components on the axes (panel A). PCoA was performed on unweighted and weighted UniFrac distances (panel B). (TIFF 14 MB) [file 40168_2014_72_MOESM5_ESM.tiff]

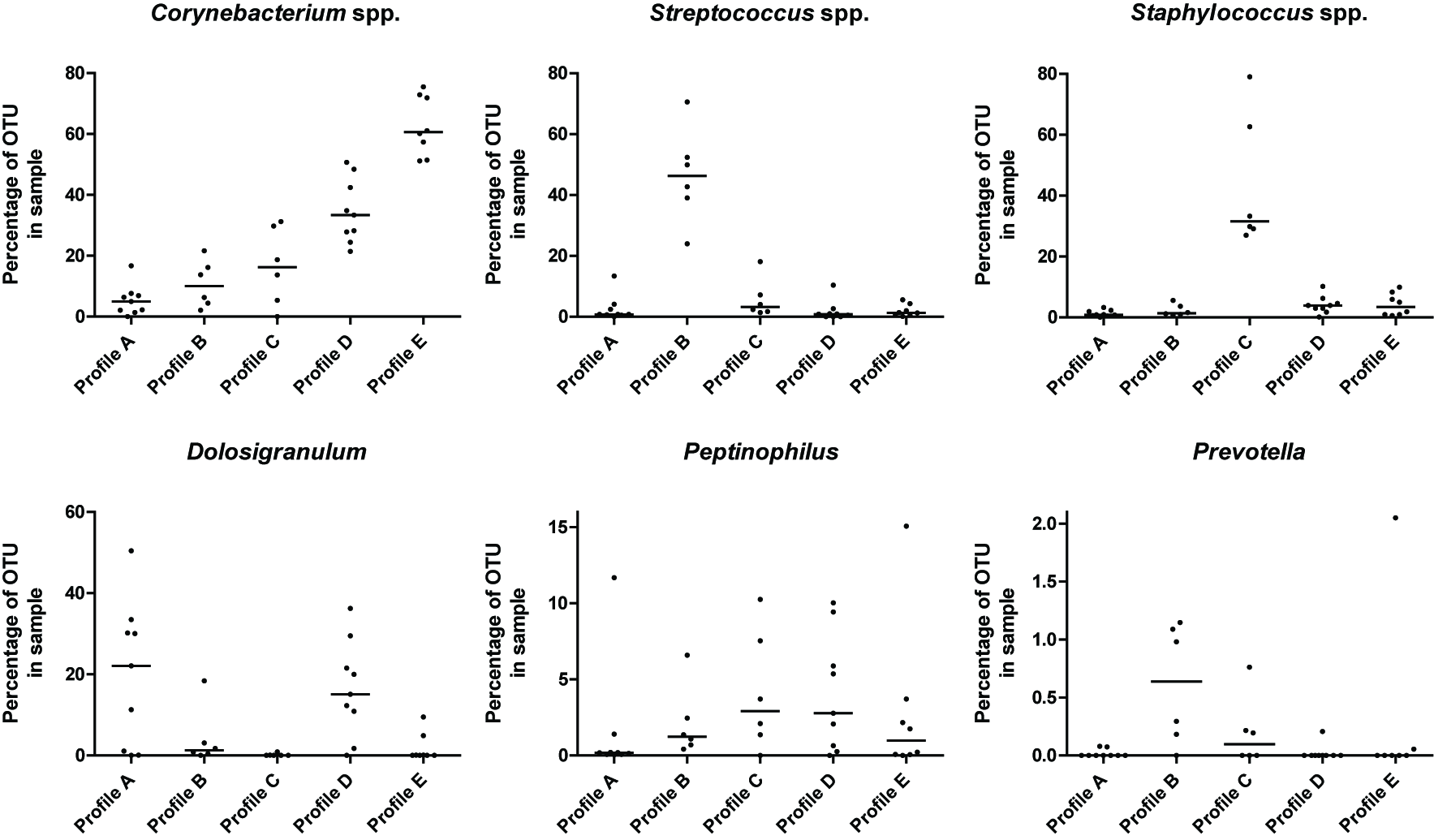

Supplement: Supplementary file 6 — Additional file 6: Figure S4: The percentage of reads from each OTU that differentiates between the microbiome profiles per NWS. NWS nasal wash sample. (TIFF 8 MB) [file 40168_2014_72_MOESM6_ESM.tiff]

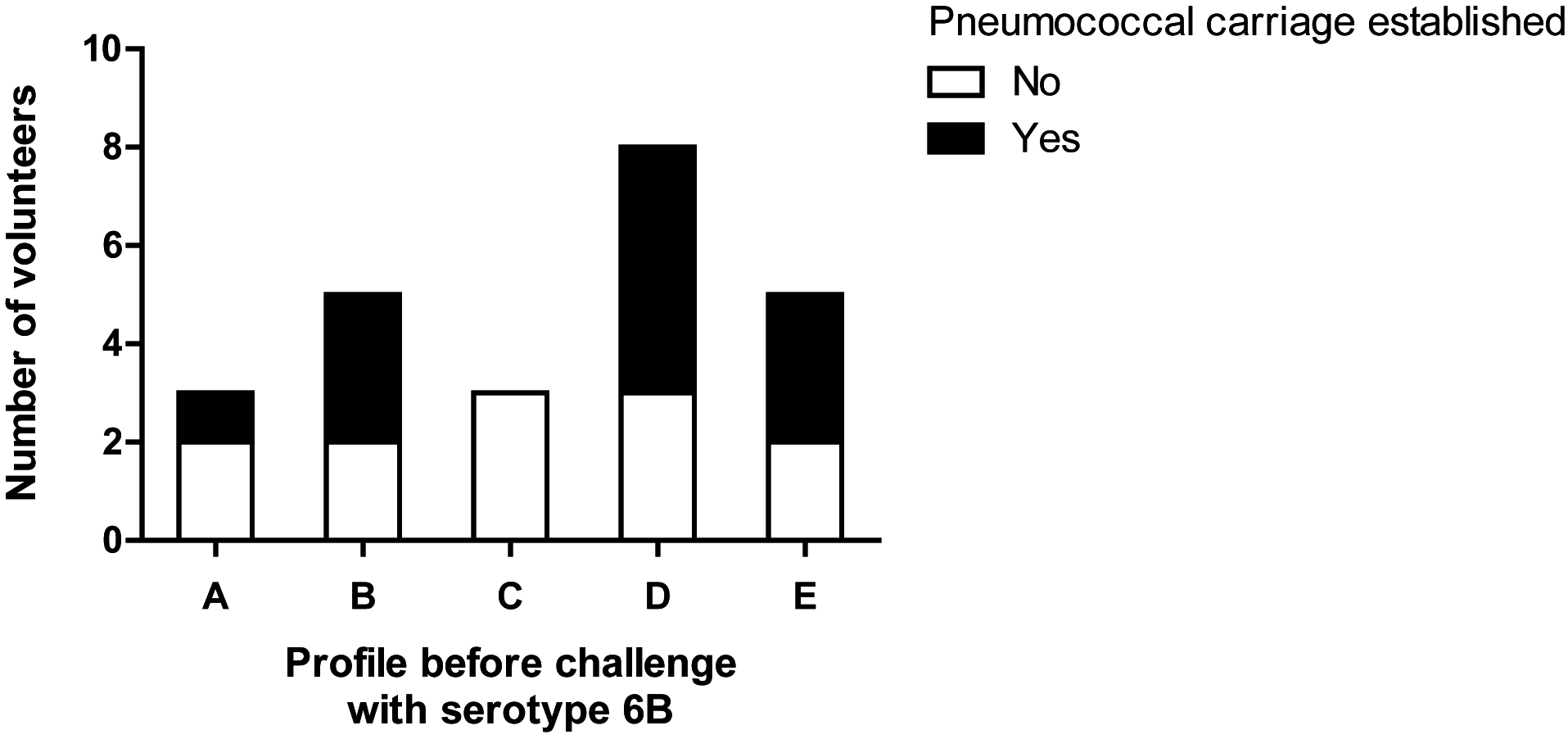

Supplement: Supplementary file 7 — Additional file 7: Figure S5: Distribution of experimental pneumococcal carriers over the five nasopharyngeal microbiome profiles at baseline. Volunteers who received a pneumococcal challenge with serotype 6B are displayed. (TIFF 7 MB) [file 40168_2014_72_MOESM7_ESM.tiff]

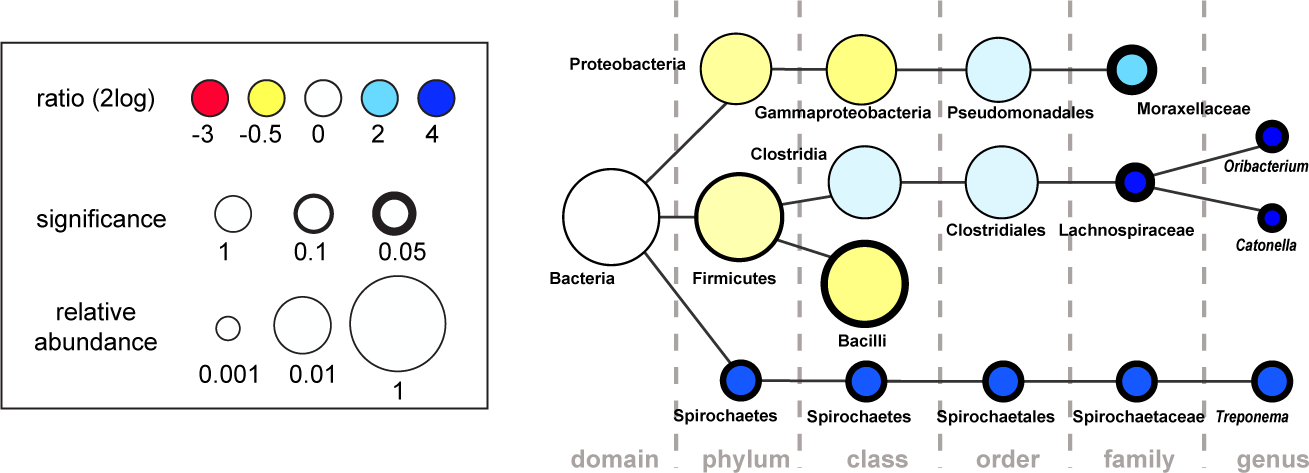

Supplement: Supplementary file 8 — Additional file 8: Figure S6: OTU abundances at baseline in volunteers with or without acquisition of experimental pneumococcal carriage. Volunteers who received a pneumococcal challenge with serotype 6B are displayed. Nodes represent taxa, and edges link the different taxonomic levels. The fold increase is calculated as the log2 of the ratio of the relative abundance in pre-existing microbiome compositions of volunteers without and with establishment of pneumococcal carriage after challenge (0 = no difference between those who did and did not establish carriage, 1 = twice as abundant in those who established carriage, and so on). The significance is expressed as the p value of a Mann-Whitney U test of the baseline samples from all challenged volunteers. Note that the relation between node size and total abundance is non-linear. (TIFF 2 MB) [file 40168_2014_72_MOESM8_ESM.tiff]

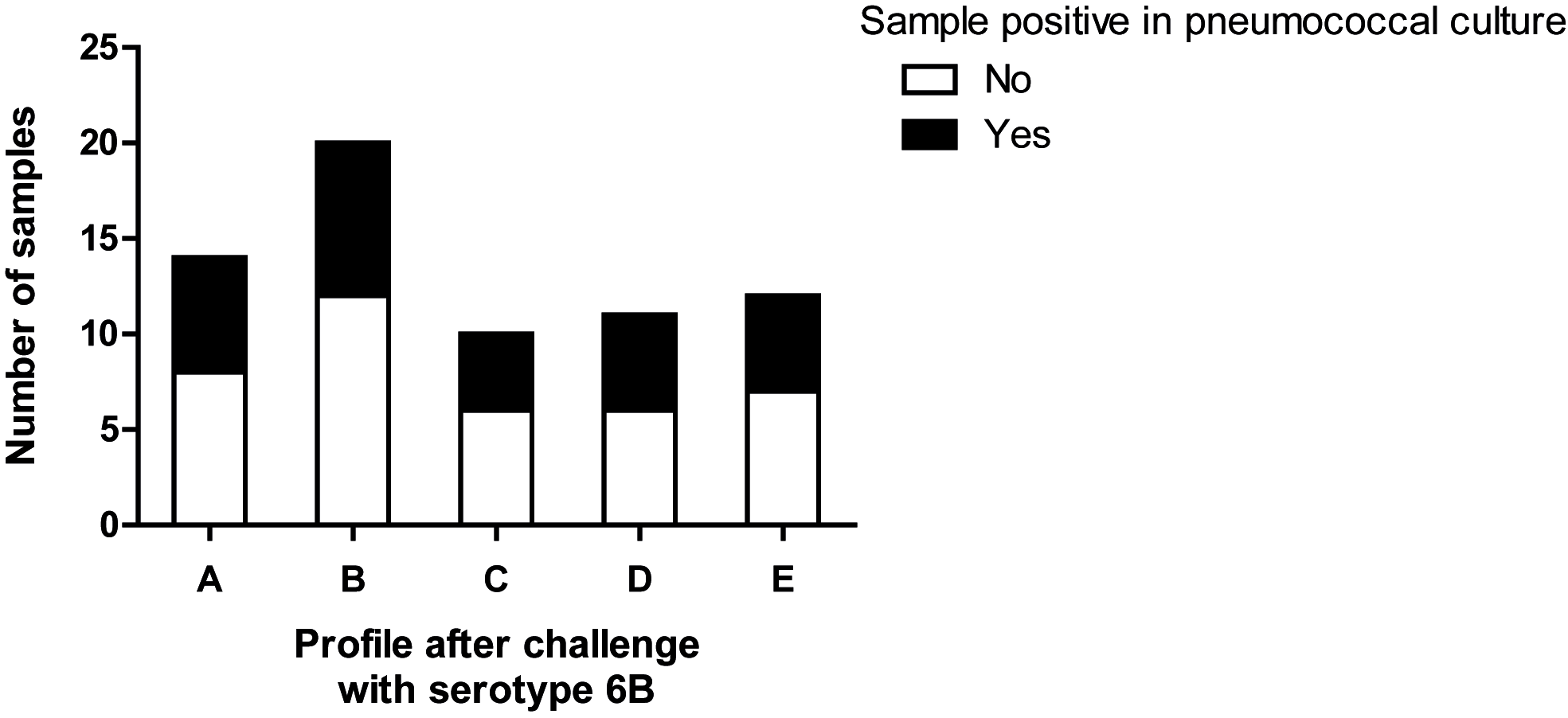

Supplement: Supplementary file 9 — Additional file 9: Figure S7: Distribution of pneumococcal culture-positive samples after challenge with serotype 6B over the microbiome profiles. (TIFF 7 MB) [file 40168_2014_72_MOESM9_ESM.tiff]

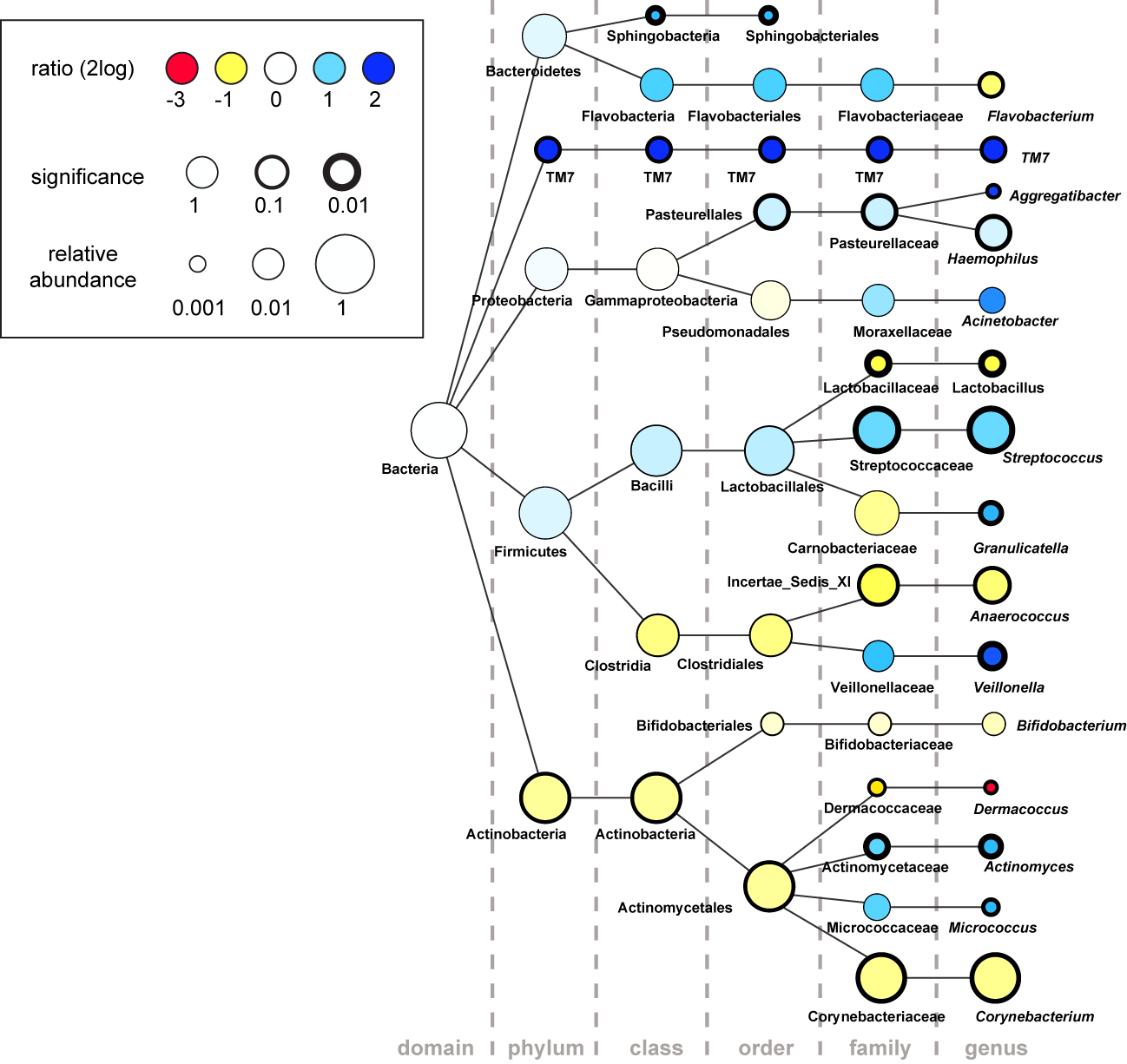

Supplement: Supplementary file 10 — Additional file 10: Figure S8: Changes from baseline OTU abundances 2 days after pneumococcal challenge among serotype 6B-challenged volunteers. Nodes represent taxa, and edges link the different taxonomic levels. The fold increase is calculated as the log2 of the ratio of the relative abundance in samples before and 2 days after pneumococcal challenge (0 = no difference between before and after challenge, 1 = twice as abundant after challenge, and so on). The significance is expressed as the p value of a Mann-Whitney U test of the samples before and 2 days after challenge. Note that the relation between node size and total abundance is non-linear. (TIFF 309 KB) [file 40168_2014_72_MOESM10_ESM.tiff]
