# Supplementary material for: The adult nasopharyngeal microbiome as a determinant of pneumococcal acquisition
Source: Microbiome. 2014 Dec 15;2:44. doi: 10.1186/2049-2618-2-44 (PMC4323220; doi:10.1186/2049-2618-2-44)
Supplement: Supplementary file 4 — Additional file 4: Table S2: Most prevalent bacterial genera present in the adult nasopharynx at baseline by natural carriage status. (DOCX 16 KB) [file 40168_2014_72_MOESM4_ESM.docx]

**Table S2. Most prevalent bacterial genera present in the adult nasopharynx at baseline by natural carriage status.**

|  |  |  | **ALL (n=38)** | | |  | **NATURAL CARRIERS (n=10)** | | |  | **NON NATURAL CARRIERS (n=28)** | | |  |
| --- | --- | --- | --- | --- | --- | --- | --- | --- | --- | --- | --- | --- | --- | --- |
| **Rank** | **Genus** |  | **Number of reads** | **% of reads** | **Relative presence n (%)^A^** |  | **Number of reads** | **% of reads** | **Relative presence n (%)^A^** |  | **Number of reads** | **% of reads** | **Relative presence n (%)^A^** |  |
| 1 | *Corynebacterium* |  | 15289 | 32.8 | 36 (95) |  | 2878 | 21.1 | 9 (90) |  | 12411 | 37.7 | 27 (96) |  |
| 2 | *Dolosigranulum* |  | 4611 | 9.9 | 27 (71) |  | 1561 | 11.4 | 8 (80) |  | 3050 | 9.3 | 19 (68) |  |
| 3 | *Staphylococcus* |  | 4256 | 9.1 | 38 (100) |  | 232 | 1.7 | 10 (100) |  | 4024 | 12.2 | 28 (100) |  |
| 4 | *Streptococcus* |  | 4219 | 9.1 | 36 (95) |  | 835 | 6.1 | 10 (100) |  | 3384 | 10.3 | 26 (93) |  |
| 5 | *Moraxella* |  | 2240 | 4.8 | 14 (37) |  | 1947 | 14.3 | 5 (50) |  | 293 | 0.9 | 9 (32) |  |
| 6 | *Haemophilus* |  | 2113 | 4.5 | 19 (50) |  | 1573 | 11.5 | 5 (50) |  | 540 | 1.6 | 14 (50) |  |
| 7 | *Porphyromonas* |  | 1813 | 3.9 | 23 (61) |  | 117 | 0.9 | 6 (60) |  | 1696 | 5.1 | 17 (61) |  |
| 8 | *Prevotella* |  | 1615 | 3.5 | 31 (82) |  | 508 | 3.7 | 7 (70) |  | 1107 | 3.4 | 24 (86) |  |
| 9 | *Peptoniphilus* |  | 1444 | 3.1 | 35 (92) |  | 362 | 2.7 | 10 (100) |  | 1082 | 3.3 | 25 (89) |  |
| 10 | *Fusobacterium* |  | 1223 | 2.6 | 19 (50) |  | 468 | 3.4 | 5 (50) |  | 755 | 2.3 | 14 (50) |  |
| 11 | *Pseudomonas* |  | 909 | 2.0 | 37 (97) |  | 217 | 1.6 | 9 (90) |  | 692 | 2.1 | 28 (100) |  |
| 12 | *Anaerococcus* |  | 880 | 1.9 | 35 (92) |  | 179 | 1.3 | 8 (80) |  | 701 | 2.1 | 27 (96) |  |
| 13 | *Propionibacterium* |  | 781 | 1.7 | 38 (100) |  | 108 | 0.8 | 10 (100) |  | 673 | 2.0 | 28 (100) |  |
| 14 | *Neisseria* |  | 236 | 0.5 | 12 (32) |  | 206 | 1.5 | 5 (50) |  | 30 | 0.1 | 7 (25) |  |
| 15 | *Finegoldia* |  | 216 | 0.5 | 28 (74) |  | 44 | 0.3 | 9 (90) |  | 172 | 0.5 | 19 (68) |  |
| 16 | *Lactobacillus* |  | 198 | 0.4 | 24 (63) |  | 51 | 0.4 | 5 (50) |  | 147 | 0.4 | 19 (68) |  |
| 17 | *Mycoplasma* |  | 155 | 0.3 | 3 (8) |  | 155 | 1.1 | 3 (30) |  | 0 | 0.0 | 0 (0) |  |
| 18 | *Treponema* |  | 111 | 0.2 | 9 (24) |  | 53 | 0.4 | 3 (30) |  | 58 | 0.2 | 6 (21) |  |
| 19 | *Campylobacter* |  | 109 | 0.2 | 15 (39) |  | 62 | 0.5 | 6 (60) |  | 47 | 0.1 | 9 (32) |  |
| 20 | *Veillonella* |  | 98 | 0.2 | 18 (47) |  | 45 | 0.3 | 5 (50) |  | 53 | 0.2 | 13 (46) |  |
| 21 | *Flavobacterium* |  | 82 | 0.2 | 23 (61) |  | 10 | 0.1 | 4 (40) |  | 72 | 0.2 | 19 (68) |  |
| 22 | *Capnocytophaga* |  | 81 | 0.2 | 12 (32) |  | 33 | 0.2 | 3 (30) |  | 48 | 0.1 | 9 (32) |  |
| 23 | *Bifidobacterium* |  | 71 | 0.2 | 14 (37) |  | 34 | 0.2 | 5 (50) |  | 37 | 0.1 | 9 (32) |  |
| 24 | *Escherichia/Shigella* |  | 68 | 0.1 | 8 (21) |  | 2 | 0.0 | 2 (20) |  | 66 | 0.2 | 6 (21) |  |
| 25 | *Gemella* |  | 67 | 0.1 | 20 (53) |  | 27 | 0.2 | 7 (70) |  | 40 | 0.1 | 13 (46) |  |
| 26 | *Streptophyta* |  | 65 | 0.1 | 21 (55) |  | 39 | 0.3 | 7 (70) |  | 26 | 0.1 | 14 (50) |  |
| 27 | *Rothia* |  | 63 | 0.1 | 21 (55) |  | 31 | 0.2 | 3 (30) |  | 32 | 0.1 | 18 (64) |  |
| 28 | *Actinomyces* |  | 48 | 0.1 | 17 (45) |  | 8 | 0.1 | 3 (30) |  | 40 | 0.1 | 14 (50) |  |
| 29 | *Granulicatella* |  | 45 | 0.1 | 8 (21) |  | 9 | 0.1 | 2 (20) |  | 36 | 0.1 | 6 (21) |  |
| 30 | *Parvimonas* |  | 40 | 0.1 | 9 (24) |  | 13 | 0.1 | 2 (20) |  | 27 | 0.1 | 7 (25) |  |

^A^Relative presence is expressed as the number of volunteers in whom ≥ 1% of reads were assigned to the genus
